# Observations on the Topography, Climate, and Disease of Tipton County, Tennessee

**Published:** 1846-08

**Authors:** R. B. Harper

**Affiliations:** Portersville, Tennessee


Art. II.—*Observations on the Topography, Climate, and Diseases of Tipton eounty, Tennessee.* By R. B. Harper, M.D., of Portersville, Tennessee.

This district of country lies under latitude 35½° N.; its western border is watered by the Mississippi; it is bounded on the north by Big Hatchie river; Beaver creek and its tributaries pass through the eastern and south-eastern parts of the county. The two last mentioned water courses have extensive low-lands bordering upon them—say from one mile to two miles wide, and besides these there are creeks of smaller size with low lands of considerable extent. The high land extends to the Mississippi on the west, forming a bluff ftom 100 to 150 feet high. The waters do not pass directly into the Mississippi, but perform circuitous routes, passing first into Hatchie and Beaver. The water courses are crooked and their currents sluggish, yet they have cut deeply into the strata, forming high banks on either side. They are subject to inundations in the winter and spring. That part of the county which borders on the Mississippi for about ten miles back, is very broken; still the land is fertile, and covered with a dense forest, which is unsubdued to a great extent, and perhaps will remain so for a long time to come. The eastern part of the county is more level, and, in the main, a good farming country, well timbered, and settled by a tolerably dense population. The low lands generally are not tillable, being for the most part too wet; and the forests upon them therefore are permitted to stand, constituting, no doubt, one of the most powerful prophylactics of our region. The country back of the large water courses is but little subject to fogs, owing perhaps to the height and density of the forests. The thermometer ranges from 9° to 94°, the temperature being very variable, frequently changing 30° in forty-eight hours, and sometimes in a single day and night. The summer sickness commences usually about the middle of July, and continues until the coming of frost, which we look for early in October. The range of the thermometer in summer is as great as 54°, having been seen as low as 40° and frequently rising to 94°. The form in which sickness generally first appears is that of a mild remittent fever, which is followed in September, or towards the latter end of August, by fevers of a more formidable type, often assuming the congestive character.

PREDISPOSING CAUSES OF FEVER.

A high temperature acts as a predisposing cause of fever by increasing the irritability of the system, and by lessening the power of vital resistance. It tends to excite excessive action in the skin and liver, by which those organs are exhausted, and thereby rendered more liable to the injurious impressions of the various exciting causes of the disease.

*Marsh-Miasma.*—From the foregoing topographical sketch it will be remarked, that all that is supposed to be necessary for the abundant production of this deleterious agent is present every season; namely, heat, moisture, and dead vegetable matter. There is at all times a sufficient quantity of moisture and dead vegetable matter. It is then only necessary that the heat be supplied to the extent necessary to produce rapid decomposition, in order to generate an abundance of miasma. And when this is done we are very certain of an incursion from summer fever. I have observed that a general rain, in the sickly season, almost invariably checks the sickness until the weather becomes dry again; and have also observed that there is less fever in a wet than a dry summer and autumn. I well remember that in the first settling of this region of country the people enjoyed much better health than they do at present; and that fevers began to abound more as the timber was extensively destroyed and the land brought under cultivation. Nor is it difficult to account for the change. All the elements necessary to the development of the fever poison were not present; there was an abundance of decaying vegetable matter, but the dense shade by preventing the solar beams from falling on it retarded decomposition so much as to prevent the evolution of malaria. A similar explanation applies to the fact that wet seasons are comparatively healthy; the excessive moisture, in that case, counteracts the formation of the effluvia which give origin to our fevers.

*Cold.*—This agent has a powerful tendency to kindle disease in subjects who have been exposed to the action of malaria. Its first effects on the system is torpor of the exhalents, and a retreat of the blood from the surface to the internal organs. The excretions are thereby retained, and the blood is surcharged with offensive matter.

PATHOLOGY OF FEVER.

The miasma having been received into the circulation by absorption, and accumulated therein, the atmosphere which is charged with this gas offers an impediment to its exit from the system. The shock is thus given by the poison to the nervous system, and the force of this is aggravated by cold which also opposes its elimination by the lungs and especially the skin.

The impression made by cold induces a suppression of the perspiratory and excretory functions, which causes the blood to recede from the surface to the internal organs, and with it the deleterious agent, which there renews the attack, and kindles into activity a disease which had been lurking in the system. The deleterious impression thus made upon the nervous system causes depression, languor, dullness, a cold creeping sensation over the body, and especially along the spinal column, general malaise, and, in some cases, severe chills and shivering. The form of fever hence arising will depend on the force of the impression, the vigor of the constitution, temperament and sensibility of the subject. Thus, other things being alike, if the impression be moderate, the stage of depression, or chill, may be of considerable length, and yet the fever succeeding a short one, passing off in a few hours by perspiration. This is a paroxysm of intermittent fever. If the impression be more powerful, the system seems to be roused to the danger, and in a short time makes a violent effort to throw off the offending agent; which effort is partly sustained until the stage of depression of the succeeding paroxysm returns. This is a paroxysm of remittent fever. Again, if the impression be still more powerful, the system is prostrated, and there is a protracted stage of depression, from which the system slowly reacts, and sometimes not all, remaining in an irritable condition until the stage of prostration of the next paroxysm is ushered in. This is a paroxysm of congestive fever.

Periodicity, or remission, is a law of the nervous system; it acts and reposes by turns, and this character is stamped upon all diseases which arise from morbid depressions made upon this system. All essential fevers exhibit this tendency, and in one type, the intermission is complete. All diseases located in the nervous system are intermittent, although the cause may be permanent. In a favorable condition of the system, several of these paroxysms of febrile phenomena may pass over without any special local inflammation. Yet in summer fevers, the liver, as well as the skin, having been previously overexcited by the solar heat, is most likely to receive the weight of the morbid impression made by the malarious poison. Its secretion is interrupted, and its circulation thereby retarded; and as the blood of the abdominal viscera passes through the liver in its return to the centre of the circulation, it is plain that engorgement will take place in them, which if not relieved will be followed by inflammation or some other lesion. The knowledge of these facts attracted the minds of medical men to the importance of exciting the secretion of the liver in the treatment of fever.

Fever, according to Eberle, cannot exist without capillary irritation, or derangement of the secretory and excretory functions. In one case, this condition being kept up for a certain period, inflammation is developed in the mucous membrane of the stomach and bowels, and becomes an independent focus of irritation, reacting upon the cerebro-spinal axis, increasing and perpetuating the morbid action; and although the secretion of the liver be restored, the fever will continue, the inflammation still existing, and having now become a cause, instead of a consequence, of congestion of the the abdominal viscera. This constitutes gastric remittent fever.

In another case, the liver being particularly predisposed, becomes irritated and inflamed, and is thus rendered less capable of performing its healthy functions, and creating a new focus of irritation, which is the condition of things existing in hepatic remittent fever.

### GASTRIC REMITTENT FEVER.

*Symptoms.*—After a premonitory stage of longer or shorter duration it commences with a chill, followed by fever, which may pass off by slight perspiration; yet on the evening of the next day a slight fever occurs, which is not entirely off at the return of the chill on the third day; which takes place generally between 8 and 12 o'clock in the morning. After a few paroxysms of this kind, usually at the commencement of the third chill, vomiting occurs, and continues till reaction is fairly established, which is often from two to four hours; and this is sometimes followed by purging. The fluid vomited mostly consists of the drinks that have been taken; but in some cases bile is thrown up. If the disease is not checked, the succeeding paroxysms may be attended with vomiting and purging of blood, and in some cases the sanguineous discharge is abundant. This variety of fever generally assumes the double tertian type, one paroxysm occurring in the morning, and the other in the evening. The tongue is often covered with a white fur, sometimes, however, with a yellow one, which, during the first few paroxysms, is moist, but afterwards becomes dry during the paroxysm: and when the disease assumes the chronic form, the white fur gives place to a dry brown coat. Sometimes this form runs into the congestive type, and when this occurs the tongue is not so dry but is still coated, and when the disease takes this course I have seen patients vomit quarts of a fluid as blue as indigo. In the commencement of this variety of febrile disease there is generally considerable pain in the head during the height of the fever, and after a few paroxysms, soreness of the stomach and bowels on pressure. The breathing is irregular during the paroxysm.

*Diagnosis.*—This form of fever is distinguished, first from the congestive, by its having a regular stage of excitement after each chill, and less sweating. The countenance and general surface are fuller than in congestive fever, and the tongue is also drier. This variety is attended with more pain, especially in the bowels, and with more acute feeling generally, than the congestive form. It is distinguished from the hepatic variety by its having a chill before every other, and, in some cases, every paroxysm, with an intermission, in some cases, and in others a distinct remission. The skin and conjunctiva of the eyes are not so yellow in this as in the hepatic type of fever.

*Treatment.*—The principal indications to be fulfilled in the treatment of remittent fever are: First, to moderate the febrile action, if it is excessive; secondly, to allay irritation, remove vitiated secretions, and, if there be a remission, or intermission, to prevent a recurrence of chill; thirdly to obviate gastro-intestinal irritation, and restore the healthy functions of the liver and alimentary canal. To fulfil the first indication, if the countenance is flushed, the pulse full, strong, firm, or corded, there can be no doubt as to the propriety of the use of the lancet; and if the venesection be performed at the commencement of the attack, if the patient be in a sitting posture, and the orifice be made large, there is no danger in permitting the blood to flow until the pulse is felt to give way perceptibly under the finger. If the circumstances just enumerated are present, there will be great benefit from the use of the lancet to the proper extent; but there are many cases in which it cannot be resorted to with advantage. I am inclined to believe that the countenance is a good guide in some cases where the pulse fails to admonish us. Where it is flushed, and the skin dry, unless the patient has been bled freely, or is very much exhausted by the disease, or is of feeble constitution, bleeding will be proper to some extent, and that extent must be determined by the physician himself.

To fulfil the second indication, after venesection, or, without it, if this be deemed unnecessary, cupping over the stomach, liver, and spleen, with or without scarification, (if the patient is very weak it is best not to scarify), will almost invariably quiet the stomach, so as to enable it to retain medicine; provided a hot foot-bath be used at the same time. If there be much vomiting my practice is to give a dose of ipecac. sufficient to operate freely two or three times, and the most suitable time for giving it I have found to be immediately after a spell of vomiting. The stomach after the operation of the ipecac. will be much more easily quieted by other remedies, among which I have used with advantage for that purpose mustard poultices applied over the stomach and liver, and suffered to remain until the skin is made very red. After this twenty drops of laudanum, with six drops of essence of peppermint, may be administered every hour until the vomiting is arrested. I have checked obstinate bilious vomiting promptly by applying a mustard plaster along the spine, and a hot foot-bath for twenty minutes, with a blanket thrown over the tub and the knees of the patient, when other remedies would not have succeeded without this adjuvant. To moderate the febrile excitement, and equalise the circulation, my plan is to give a teaspoonful of spirits of nitre every two hours, and sponge the extremities with equal parts of warm vinegar and water, or weak lye also warm, as often as may be necessary, say every hour, or once in two, three, or four hours. This hastens the sweating stage, which terminates the paroxysm. These applications should be made freely and often, where they are necessary, and in bad cases should be extended over the whole body. After the irritation is sufficiently allayed, I give powders composed of calomel grs. vi, Dover's powder grs. vi, every three hours, until three or four have been taken. If they do not operate sufficiently in three hours after the last is taken, I give oil to assist the operation. If the medicine operates more than five times freely, it should be checked by laudanum or opium. If the chill should return while the medicine is operating, it will aggravate the symptoms of the succeeding paroxysm; the operation therefore should always be checked before the chill is expected. This course should be followed up every thirty-six hours, until the secretions become free and healthy, especially from the liver and alimentary canal, or until the fever is checked by means to be used during the intermission. When the patient has once been salivated, or his system is easily affected by calomel, its effects should be closely watched, and never carried to the extent of producing ptyalism, if it can be avoided; for calomel produces all its good effects in fever short of this condition. I am partial to the blue mass in patients who are easily salivated, or who have been severely mercurialized, as I believe it is not so apt to excite the state called dry salivation, or to produce eating ulcers in the mouth. This substance, or calomel given in small and repeated doses, until a sufficient quantity is given, and worked off when the system is freest from irritation, while its effects are fully ascertained before more is given, produces a much better effect than when given in large doses. The successful operation of the medicine depends upon the promptness with which the irritation of the stomach and bowels is allayed; and I have found no medicine to answer this purpose better than opium. The injury said to be produced by it upon the brain, I have never seen follow its use under the above circumstances. The delirium is no greater after, than before it was given; but the ease experienced by the patient is almost indescribable. The torpor of the bowels, feared from its use, does not follow when given as directed above. It is only to be given where it is necessary to restrain the action of the bowels, and this is to be done at any time when they are too active, regardless of fever. I very often use pulv. Doveri and calomel in small doses, (say one or two grains each, and if necessary add half a grain of opum), to be given every hour or two. I am partail to this form of opiate on account of its tendency to act on the skin.

In recent cases, and all others at any stage, when there is no positive indication of inflammation of any important internal organ, during the remission, which is followed by a chill, I give quinine v grains, pulv. Doveri and camphor each iii grains, in a powder, every three hours, commencing seven hours before the chill, and continuing until five are given; this course should be pursued before each chill; and in cases where there is not sufficient time to give and work off the purgative medicine in time to anticipate the chill with the tonic powders, I have combined the calomel, quinine and camphor. After the time for the chill has passed I give oil to work the calomel off. The quinine in the tonic powders may be increased when the case requires it, or the composition may be varied to suit each particular case. I have thought that the camphor not only acted as a narcotic stimulant, but modified the tendency of the quinine to act upon the brain. This course should be pursued before each chill, until the system is clear of disease, unless the case becomes complicated by inflammation.

In cases where the tenderness of the stomach and bowels on pressure, the dry tongue, and the excitement, continued from chill to chill, contraindicate the use of quinine, I give a powder composed of camphor and pulv. Doveri each iii grs., calomel iv grs., every three hours, commencing seven hours before the chill, until four are given, and in two hours afterwards give oil to work them off. This course should be repeated before the next chill; and in addition a mustard plaster applied the whole length of the spinal column one hour before the chill, and allowed to remain until the skin is very red, exerts a happy influence. The mustard employed in this way has a powerful tendency to prevent a chill, and even to stop it when it is on the patient, by communicating its stimulating influence to the heart and arteries. I have seen the chill arrested in fifteen minutes by its application when the patient had been shaking severely for some time.

Third. When the above named symptoms of tenderness of the stomach and bowels, dry red tongue, &c., are present, great benefit will be received from cupping freely and frequently over the abdomen, after each application having a poultice thinly spread, and sprinkled with mustard, applied so as to keep the skin red and moist. This retains the blood upon the surface and thereby relieves the internal organs. The sponging spoken of above should also be freely employed. Gum water or slippery-elm water is the most eligible drink, and may be used cool. Nor is a moderate use of plain water objectionable, although the patient may be taking calomel. I have never seen any bad effect from its use and my practice is to allow my patients to use cold water freely. No drink in my opinion is equal to it in a hot burning fever, and so far from being prejudicial, it will often facilitate the favorable operation of calomel, while, most assuredly, according to my experience, it has no tendency to bring about salivation.

If the disease seems likely to assume a chronic form, a pill composed of six grains of blue mass, one grain of opium, and two grains of ipecac., may be given every three hours until four have been taken, followed in three hours by a dose of oil, with twenty drops of spirits of turpentine, unless the bowels are moved sufficiently without it. This course should be repeated every thirty-six hours, until the secretions become healthy, and the tenderness is relieved. It is beneficial to use the plaster to the spine every twelve hours or oftener, and the cupping and poultice as directed above. If the symptoms do not improve under this treatment in thirty-six or forty-eight hours, a large blister should be applied, after cupping, over the soreness; and if the head is affected the back of it should be shaved, as high as the occipital protuberance, and a blister four inches wide laid across from ear to ear. If the patient is not much exhausted, and the head hot, he may be scarified and cupped behind the ears, before the blister is applied. Mustard plasters of large size may be applied to the calves of the legs freely, and a blister on the inside of each thigh, above the knee. I think this a much better situation for blisters than the ancles, for there is not the same danger of producing troublesome sores when applied to the former. When bloody purging attends, opium and calomel, with the external applications directed above, given in two grain doses each every two hours, the opium being increased or decreased according to circumstances, will be found to accomplish all that may be expected ot medicine. It is scarcely worth while to speak of the extent to which this medicine should be pushed. Medicine should be limited by the effect, and not by the quantity. If it is given in broken doses, and time allowed for the previous dose to have its effect, before another is given, there is no danger. When the bowels are checked they should not be moved under twenty-four hours, and then it is preferable to use the syringe.

### THE CONGESTIVE VARIETY OF BILIOUS REMITTENT FEVER.

This is a more fatal form of fever than the preceding, though the type is the same, except that the chill oftener precedes the fever every day than in the former. The chill continues from one to twelve hours before the excitement follows, and sometimes reaction never takes place. In some cases the first chill differs from the ordinary intermittent only in being more protracted, and the system remaining more prostrated; it is therefore very important that the practitioner who is not acquainted with this form of fever should be on his guard, for the patient may die in the next regular paroxysm. The excitement which follows the stage of depression is not, generally, very great; the extremities remain cold, and the surface shrunk; and in most cases there is a copious perspiration exuding from the skin, warm when the skin is warm, and cold when it is cold. Sometimes this perspiration only appears when the sickness is great. Sometimes the stomach and bowels are irritable. When vomiting is an attendant, it is more aggravated during the paroxysm. The fluid vomited consists mostly of the drinks taken; the operations from the bowels are large and watery; the countenance is ghastly and expressive of anxiety; the lips generally blue. The eyes are sunken; the tongue is moist, flabby, and not very thickly furred. The senses are generally not much affected, but occasionally the patients become delirious as soon as the fever rises, and sink into a state of stupor, from which it is difficult to rouse them until the paroxysm is over. In such cases there is less purging and less vomiting, till the fever begins to subside. The breathing is frequently performed by a long deep sigh; and when stupor prevails the breathing is more laborious still. The patient is generally restless, but does not suffer much pain.

We have a great many cases which vibrate between this and the preceding variety. It is only necessary in these to modify the treatment according to circumstances.

*Diagnosis.*—In this varety, the chill is more protracted; the excitement which follows is not so great, or so regular; the surface is more shrunk; the extremities are much cooler; and the perspiration is more profuse. The tongue is also moister than in the preceding variety. It is distinguished from the hepatic variety by there being in it a more general perspiration, less excitement, a marked state of intermission, or remission, and a general sunken appearance of the countenance.

*Treatment.*—In this variety the three indications are:—First; to allay the irritation of the stomach and bowels. Secondly; to moderate the excitement where this exists, or if reaction be not established to promote it; and thirdly, to prevent the recurrence of the paroxysm.

To fulfil the first, the same remedies prescribed in the preceding variety may be employed; viz: an emetic; and the vomiting over, laudanum and essence of peppermint, cupping, mustard plasters, followed by a cloth wet with laudanum to the stomach and bowels, and to the spine and legs; a strong warm salt-water enema, followed after it is discharged, by a cold one made mucilaginous by slippery elm.

2d. After the irritation is sufficiently allayed, a powder composed of pulv. Doveri and camphor, each three grains, and calomel six grains, may be given every three hours until four are taken, and if the stomach still be irritable opium may be added to the prescription. In cases where the intermission is not sufficiently long to admit the use of quinine as heretofore directed, five grains may be added to the powders which should be given at such times that the last would be taken one hour before the chill. Cathartics are to be used sparingly in this form of fever. but if the bowels are not moved in six hours after the last powder is given, oil should be taken with twenty-five or thirty drops of spirits turpentine; or an enema may be used, and the oil postponed until after the chill has passed, together with its accompanying irritation. I combine camphor with all the calomel I give in this form of fever. If the powders cannot be borne with the quinine, that article may be left out, and the powders given without it; but this will rarely be necessary, where the case is one indicating the use of the quinine. When the reaction is broken, it is well to rub the surface with warm vinegar and cayenne, and strong salt-water or tincture of camphor, alternately. The rubbing should be resorted to every hour, and should be brisk, done under the bed-clothes, and diligently kept up until reaction is fairly established. In addition to the above, I apply the mustard plaster to the spine and legs, as directed in the former variety. It has a powerful effect in rousing the nervous system. But if the system should sink into a complete state of collapse, or should it be in that state when the physician is called, the cold douche should be resorted to; and if reaction does not take place in an hour and a half, may be repeated. This will sometimes succeed when nothing else will, and should never be neglected in cases demanding it.

3d. To fulfil the third indication, I use the same means recommended for this purpose in the preceding variety, with an increase of the quinine in the powders to eight or ten grains. These powders should be commenced as soon as the intermission or remission is sufficiently distinct. The powders should be given every three hours, and so timed that one will be given one hour before the chill, and if the chill should return sooner than it was expected, a portion should be given as soon as it is felt. In these cases we are not to wait for all the favorable indications for giving quinine laid down by the older writers; but are to risk everything else rather than the chill. It will be found that there are but few cases in which tonics are necessary that the powders will not be retained, provided the collateral treatment, heretofore spoken of, be attended to. I often add calomel to the powders when the secretions are disordered. This course should be pursued in the intervals unless something should contraindicate it; such as a stomach too irritable to retain the medicine, severe pain in the stomach or head, &c. In such cases, which are but few, the camphor powders mentioned above should be substituted.

When patients sink into stupor, I use the same means directed for this condition in the preceding form of fever, with the addition of stimulating enemata every three or four hours. I am well satisfied that they act as powerful revulsives upon the brain in cases of this kind. After the patient becomes convalescent, he should take an occasional blue-pill and some tonic, for some time, such as Peruvian bark, columba, gentian, and rhubarb in wine, &c., to prevent a relapse, which is very apt to take place both in this and the preceding variety of fever, without this precaution.

### HEPATIC VARIETY OF REMITTENT FEVER.

*Symptoms.*—This variety generally comes on with feelings of languor and dullness which last for several days before the attack is fully evident. The next stage is ushered in by chilliness, pain in the head, back, and extremities, which symptoms are soon followed by high fever, strong pulse, flushed countenance, severe pain in the head, and sickness at the stomach. These continue, with slight remissions in the mornings and evenings, for several days without any symptom of chill. Great restlessness attends, with general aching, intense thirst, and furred tongue, but the fur in a few days becomes brown and dry in the centre.

This form is generally easily managed in the commencement, and is treated successfully by a variation of the treatment laid down for the former varieties; adapting the treatment to the symptoms. Bleeding, cupping, mustard, and blisters, calomel, spirits nitre, acetate of ammonia, sponging, slippery elm water, and cold water, and quinine, &c., in the last stage, if admissible, constitute the proper means.

There is a condition in the last stage of this fever in which calomel will fail to excite the action of the liver, unless assisted by other means. In such cases it will be found that extensive irritation of the surface by mustard, a mustard plaster along the spinal column, and camphor, and if necessary Dover's powder combined with calomel, will bring the system into a condition to receive the impression of the mercurial.

April 22d, 1846.

